# Chrysin Pretreatment Improves Angiotensin System, cGMP Concentration in L-NAME Induced Hypertensive Rats

**DOI:** 10.1007/s12291-018-0761-y

**Published:** 2018-06-14

**Authors:** Ramanathan Veerappan, Thekkumalai Malarvili

**Affiliations:** 1 Research Department of Biochemistry, Enathi Rajappa Arts and Science College, Pattukkottai, Thanjavur, Tamilanadu India; 2 Bharathidasan University Constituent College, Orathanadu, Tamilnadu India

**Keywords:** Angiotensin-II, Chrysin, Hexo oxygenase, Left ventricular function, Nitric oxide, Renin-angiotensin system

## Abstract

N^ω^-nitro-l-arginine methyl ester (L-NAME) is a non-specific nitric oxide (NO) synthase inhibitor, commonly used for the induction of NO-deficient hypertension. The objective of the present study was to investigate the effects of chrysin with flavnoids, on L-NAME-induced hypertensive rats. Methods: An experimental hypertensive animal (180–220 g) model was induced by L-NAME intake on rats. In treatment chrysin was orally administered 25 mg/kg body weight (b.w.). Blood pressure was measured by tail cuff plethysmography system. Cardiac and vascular function was evaluated by Langendorff isolated heart system with Angiotensin II (Ang-II), Hexo oxygenase (HO-1), cyclic guanosine monophosphate (cGMP) concentration in tissues respectively. Rats with hypertension showed an elevated blood pressure (BP), left ventricular functions, ang II, and decreased cGMP concentration of tissues. Treatment of chrysin is reverse to near normal in left ventricular functions, Ang-II, Ho-1 and decreased cGMP concentration of tissues. The antihypertensive effect of chrysin appears to be mediated by a reduction in left ventricular functions, cardiac oxidative stress and Ang-II, an increase in cardiac HO-1, cGMP concentration and a prevention of plasma nitric oxide loss.

## Introduction

Hypertension and its related conditions as like as coronary artery disease, stroke, heart failure, and chronic renal failure is a growing public health issue for which successful treatment often remains inadequate [1]. Cardiovascular diseases including hypertension are often associated with behavioural alterations. Hypertension is an over whelming global challenge, which ranks third as a means of reduction in disability-adjusted life-years [2]. Hypertension has been affected much more than 600 million peoples and due to final results in 13% of total deaths world widely, and it also estimated that 29% of the world’s adult will have hypertension by 2025. Reactive oxygen species (ROS) also have been shown to be critical determinants in hypertension. The contribution of oxidative stress to the pathogenesis of hypertension is supported to rely upon inactivation of the NO [3]. Renin–angiotensin system (RAS) plays a vital role in the regulation of vascular function and blood pressure [4]. Chronic NO inhibition with N^ω−^nitro-l-arginine methyl ester (L-NAME) can increase regional vascular resistance, raise the blood pressure, and oxidative stress accompanied with renal damage. The primary function of nitric oxide is the regulation of vascular tone, inhibition of vascular smooth muscle cells proliferation and platelet aggregation [5] which makes chronic inhibition of basal nitric oxide with an orally active nitric oxide synthase (NOs) inhibitor (L-NAME), a particularly interesting model of hypertension. Isoforms of the nitric oxide synthase are divided in three types namely neuronal (nNOS), inducible (iNOS) and endothelial (eNOS) [6]. Nitric oxide (NO) is a important regulator of vascular endothelial function and blood pressure. The chronic administration of nitric oxide synthase inhibitors provides an animal experimental model of hypertension [7].

**Fig. 1:**
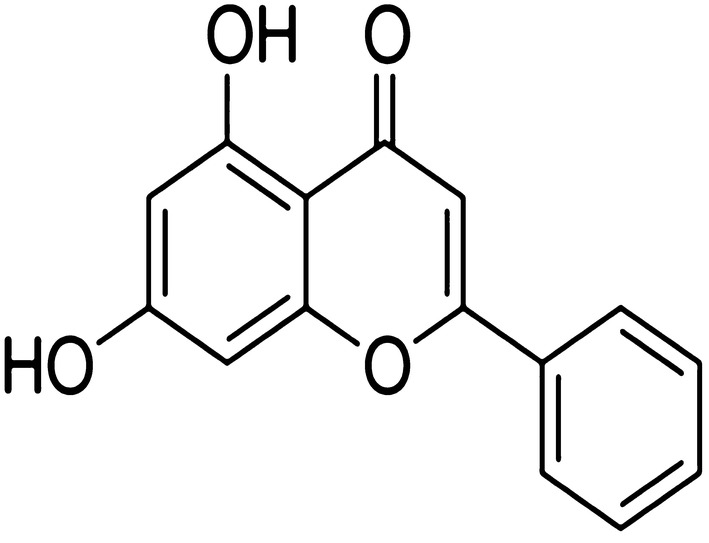
Chemical structure of chrysin (5,7 dihydroxyflavone)

Ang II induced hypertension was associated with ascending superoxide production of vascular smooth muscle cells, through the activation of membrane-bound NADPH oxidase [8]. The NO stimulates guanylyl cyclase (sGC), which is responsible for converting guanosine triphosphate to cyclic guanosine monophosphate and playing an important role in activating of cGMP dependent protein kinase and resulting vascular smooth muscle relaxation [9]. Flavonoids are plant polyphenolic compounds that consist of a number of classes, such as flavanols, flavones and flavans. Chrysin (5,7-dihydroxy flavones structure shown in Fig. 1) a naturally occurring flavones which may contained in flowers such as the blue passion flower (*Passiflora caerulea*) and the Indian trumpet flower, and in edible food items such as mushroom [10], honey and propolis [11]. Chrysin found to contain antioxidant, anti-allergic, Anti-inflammatory, antiestrogenic, anxiolytic [12] and antihypertensive [13] properties. At the same time chrysin has been found to posses tyrosinase inhibitory activity and moderate aromatase inhibitory activity. It can also inhibit estradiol-induced DNA synthesis [3]. A number of its derivatives have been prepared, which helps in improving the biological activity of chrysin [14]. C-iso-prenylated hydrophobic derivatives of chrysin are potential P-glycoprotein modulators in tumour cells [15]. The previous study showed that chrysin has antihypertensive effects, which reduces hepatic, renal damages and endothelial dysfunction in L-NAME-induced hypertensive rats [16]. The present study is aimed to evaluate the effect of chrysin on left venricular functions, cGMP concentration, Ang II and HO-1 in L-NAME-induced hypertensive rats. The findings were compared with those control and unsupplemented groups.

## Materials and Methods

### Chemicals

Chrysin and L-NAME was purchased from Sigma Chemical Co. (St. Louis, MO, USA). All other chemicals used in this study were of analytical grade and obtained from E-Merck or HIMEDIA, Mumbai, India.

### Animals

All the animal handling and experimental procedures were approved by the Institutional Animal Ethics Committee of Bharathidasan University (Registration no: 418/01/a/date 04.06.2001) and animals were cared for in accordance with the Indian National Law on Animal Care and Use. Male Wistar rats (180–220 g) were purchased from the Indian Institute of Science, Bangalore, India. Rats were housed in plastic cages with filter tops under controlled conditions of a 12 h light–dark cycle, 50% humidity and temperature of 28 °C. All rats received a standard pellet diet (Lipton Lever Mumbai, India) and water ad libitum (BDU/IAEC63/2013).

### Blood Pressure Measurements

Systolic and diastolic blood pressures were determined by the tail-cuff method (IITC, model 31, Woodland Hills, CA, USA). The animals were placed in a heated chamber at an ambient temperature of 30–34 °C for 15 min and almost nine blood pressure values were recorded for each animal. The lowest three readings were averaged to obtain a mean blood pressure. All recordings and data analyses were done using a computerized data acquisition system and software.

### Induction of L-NAME-Induced Hypertension

L-NAME (40 mg/kg B.W) was dissolved in drinking water and given to rats at regular interval of 24 h for 8 weeks. Mean arterial blood pressure (MAP) was measured using tail cuff method. MAP measurements were performed at the time of 1–8 weeks [16].

### Study Design

Animals were divided into four groups of six rats each and all were fed with the standard pellet diet. The rats were grouped as given below.

Group I: Control
Group II: Normal + chrysin (25 mg/kg of B.W) after 4th week
Group III: L-NAME induced hypertension (40 mg/kg of B.W)
Group IV: L-NAME induced hypertension + Chrysin (25 mg/kg of B.W).

Chrysin (25 mg/kg of B.W) was administered orally in the morning once in a day for 4 weeks and Chrysin (25 mg/kg of B.W) dose was taken for this study based on previous our studies [16]. The compound was suspended in 2% dimethyl sulfoxide solution and fed by intubation. After the 8th week morning, the animals were sacrificed by cervical dislocation. The blood was collected in clean dry test tubes and allowed to coagulate at ambient temperature for 30 min. The blood, collected in a heparinized centrifuge tube, was centrifuged at 2000 rpm for 10 min and the plasma separated by aspiration was used for estimations.

## Experimental Methods

### Langendorff Isolated Heart Study

The left ventricular function of the rat heart was evaluated using the Langendorff heart preparation. After anesthesia, the heart was excised and placed in cooled (4 °C) Krebs–Henseleit bicarbonate solution [a composition (in mM): 118 NaCl, 4.7 KCl, 1.2 MgSO4, 1.2 KH_2_PO_4_, 2.3 CaCl_2_, 25.0 NaHCO_3_, 11.0 glucose]. The heart was then attached to the cannula through aorta and retrogradely perfused with the Krebs solution maintained at 37 °C and continuously gassed with a mixture of 95% O2–5% CO_2_. Perfusion pressure was kept constant at 80 mmHg. Isovolumetric recordings of rate of pressure development (+dp/dt) and rate of decline pressure (2dp/dt) were obtained from a balloon catheter inserted into the left ventricle. The ventricular balloon was connected via fluidfilled tubing to a pressure transducer (AD Instruments, Australia) for continuous assessment of ventricular performance as described previously [17].

### Heart NADPH Oxidase Assay

Nicotinamide adenine dinucleotide phosphate (NADPH) oxidase activity measured as superoxide production in the cardiac samples was based on a reduction of ferricytochrome c in ferrocytochrome at pH 7.8 as described by Mustapha et al. [18]. The samples were homogenized in 0.25 M sucrose (pH 7.8). The reaction mixture containing 250 mg/l cytochrome c, 100 mM NADPH and 50 mg of protein samples was incubated at 37 °C for 120 min, either in the presence or absence of diphenyleneiodonium (DPI, 100 mM). The absorbance of the reduction of cytochrome c was taken at 550 nm. Superoxide production was calculated from the difference between the absorbance of the samples incubated with and without DPI using an extinction coefficient of 21 mM-1 cm-1.

### Quantification of Angiotensin II Levels

Cardiac tissue was homogenized in a 5 mM potassium phosphate buffer, (pH 7.4 containing 0.9% sodium chloride and 0.1% glucose) along with the protease cocktail mentioned in Sect. 3.2.6 and centrifuged at 10,000×*g* for 15 min at 4 °C. The supernatant was then collected and the protein was determined using the Bradford method. Plasma was collected at the time of sacrifice, using EDTA (1.75 mg/ml) and centrifuging for 10 min at 3000×*g*. Subsequently, angiotensin II concentration was quantified by enzyme immunoassay (EIA), (Cayman chemical Company, Ann Arbor, MI, USA). Using a fixed amount of monoclonal antibody the assay is based on competition between angiotensin II and a tracer (acetylcholinesterase-labelled monoclonal antibody (mAb)). A standard curve was established using known concentrations of angiotensin II which had been previously extracted using methylene chloride. Standards were obtained from the absorbance recorded at 412 nm with a microplate reader (SpectraMax 340PC, Molecular Device, CA, USA). Plasma angiotensin II levels were calculated (pg/ml). In the case of cardiac tissue, angiotensin II levels were then standardized using sample protein content, (pg/mg of protein) [19].

### Measurement of Cyclic Guanosine Monophosphate (cGMP)

The aortic and cardiac tissues homogenates were resuspended separately in 5% trichloro acetic acid (TCA) and were centrifuged at 1500×*g* for 10 min at 41 °C. The supernatant was extracted four times with 1 ml of water-saturated ether and the extract was dried and cyclic GMP levels were measured by using cGMP EIA kit obtained from Cayman Chemical Company, USA.

### HO-1 Assays

Heme oxygenase (HO-1) (Enzo Life Sciences, MI, USA) activity was analyzed in cardiac samples using commercially available kits following the manufacturer’s instructions. The colored end products of this enzyme was measured by a microplate reader (Molecular Devices, Sunnyvale, CA, USA) at 450 nm [20].

### Statistical Analysis

Statistical analysis were analysed by one-way analysis of variance (ANOVA) followed by Duncan’s multiple range test (DMRT) using a commercially available Software Package for the Social Science (SPSS) software package version 11.0. Results were expressed as mean ± S.D. for six rats in each group. For all the statistical tests, values of *P* < 0.05 were statistically significant [21].

## Results

Table 1 shows cardiovascular function—Langendorff and organ bath study. The systolic contractility of the isolated heart was measured by the first temporal derivative of the left ventricular pressure (LVP) positive development (+dP/dt, in mmHg/s), and the isovolumetric relaxation was measured by the first temporal derivative of the LVP negative pressure (− dP/dt, in mmHg/s). In the heart of LNAME rats, the rate of LV pressure (+dP/dt, in mmHg/s) and the rate of LV pressure decline (− dP/dt, in mmHg/s) was significantly (*P* < 0.05) reduced. Supplementation chrysin significantly (*P* < 0.05) promoted ventricular function in L-NAME rats.

**Table 1:** Effect of chrysin on left ventricle functions of control and L-NAME induced hypertensive rats

	Control	Control + 25 mg chysin	L-NAME induced hypertension	L-NAME + 25 mg chrysin
LVDP (mmHg)	90.01 ± 7.10^a^	89.57 ± 7.12^a^	124.19 ± 9.76^b^	93.04 ± 7.09c
LVEDP (mmHg)	3.44 ± 0.26^a^	3.36 ± 0.27^a^	7.8 ± 0.35^b^	3.50 ± 0.29^c^
Maximum +dP/dt, mmHg/s	2282 ± 106^a^	2298 ± 102^a^	1762 ± 122^b^	2246 ± 148^c^
Minimum −dP/dt, mmHg/s	1972 ± 72^a^	1986 ± 74^a^	1509 ± 95^b^	1960 ± 98^c^

Values are mean ± S.D. for six rats in each group

Values not sharing common superscript are significant with each other at *P* < 0.05 (Duncan’s multiple range test)

**Fig. 2:**
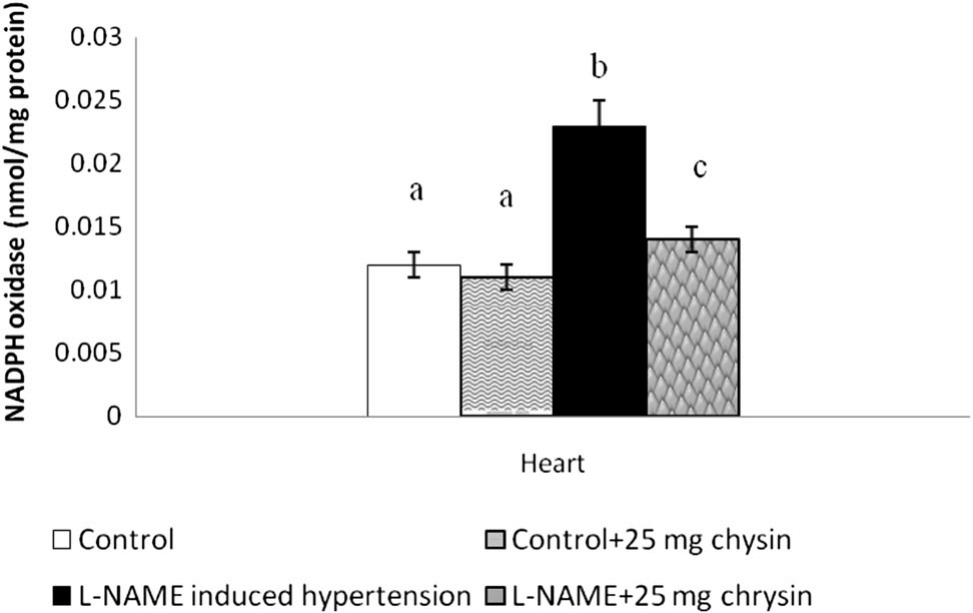
Effects of chrysin on NADPH oxidase enzyme activity in L-NAME hypertensive rats. Columns are mean ± S.D. for six rats in each group. Columns not sharing common superscript are significant with each other at *P* < 0.05 (Duncan’s multiple range test)

A significant elevation of cardiac NADPH oxidase activity was observed in the L-NAME group. Supplementation of chrysin group IV displayed lower enzyme activity as compared to group IV. No significant difference was noted among the groups I and II (Fig. 2).

**Fig. 3:**
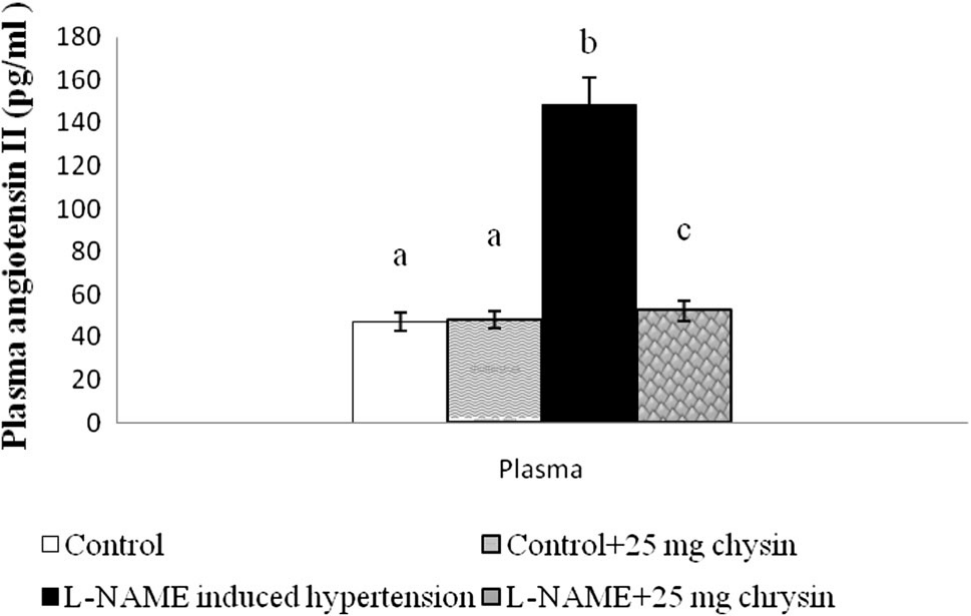
Effects of chrysin on Plasma angiotensin II in L-NAME hypertensive rats. Columns are mean ± S.D. for six rats in each group. Columns not sharing common superscript are significant with each other at *P* < 0.05 (Duncan’s multiple range test)

Figure 3 shows as an increase in plasma Ang II was found in L-NAME hypertensive rats compared to the level in control rats. Administration of chrysin reduced plasma Ang II level compared to untreated rats (*P* < 0.05). There is no significant change between group I and II.

**Fig. 4:**
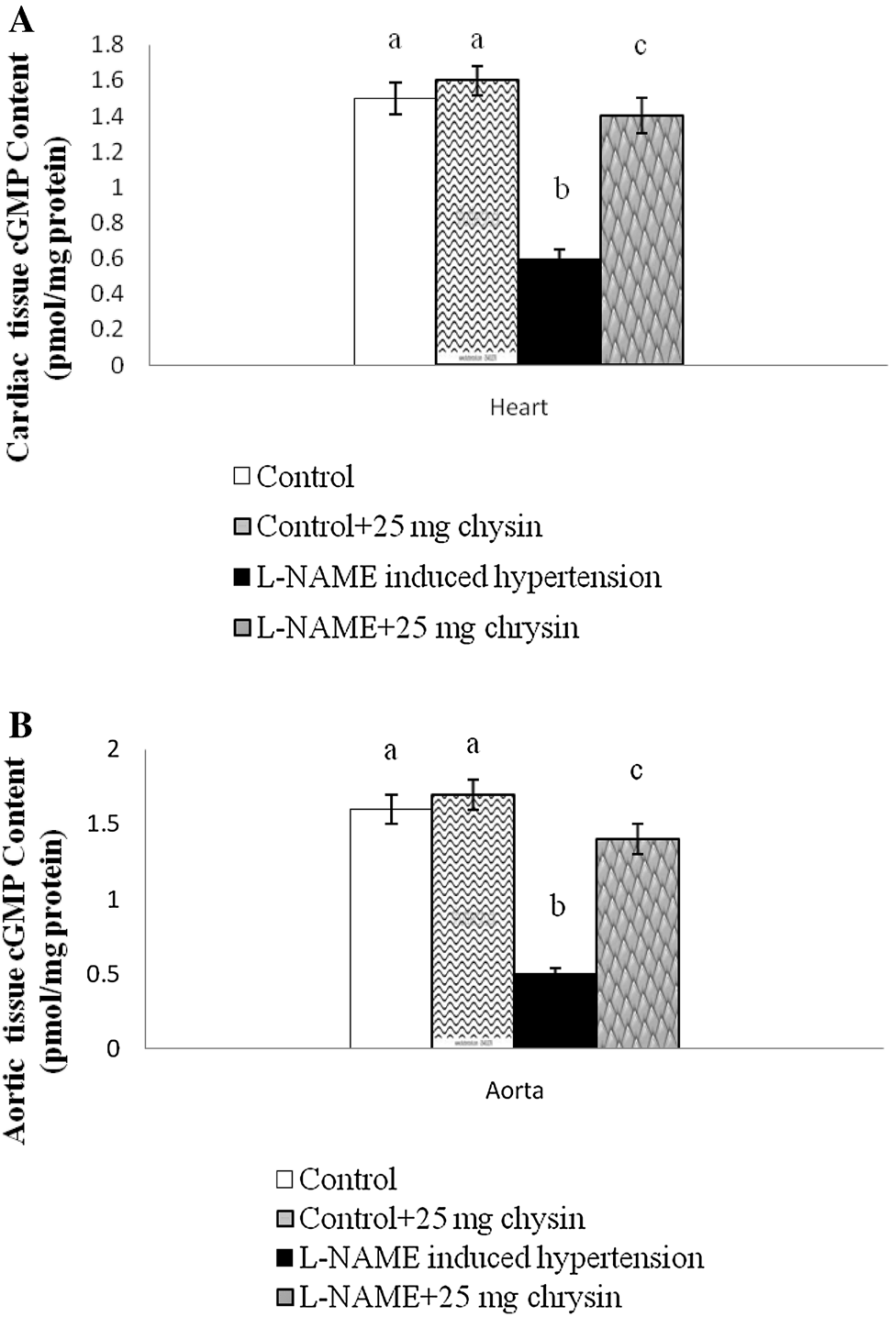
**a** Effects of chrysin on Cardiac cGMP content in L-NAME hypertensive rats. Columns are mean ± S.D. for six rats in each group. Columns not sharing common superscript are significant with each other at *P* < 0.05 (Duncan’s multiple range test). **b** Effects of chrysin on Aortic cGMP content in L-NAME hypertensive rats. Columns are mean ± S.D. for six rats in each group. Columns not sharing common superscript are significant with each other at *P* < 0.05 (Duncan’s multiple range test)

Effect of chrysin on the cyclic guanosine mono phosphate (cGMP) level Fig. 4(a, b) shows the cGMP level in cardiac and aortic tissues of L-NAME induced hypertensive rats. The levels of cGMP decreased significantly in L-NAME rats, and the administration of chrysin significantly increased the level of cGMP and the effect was more pronounced at 25 mg/kg dose. There is no significant change between group I and II.

**Fig. 5:**
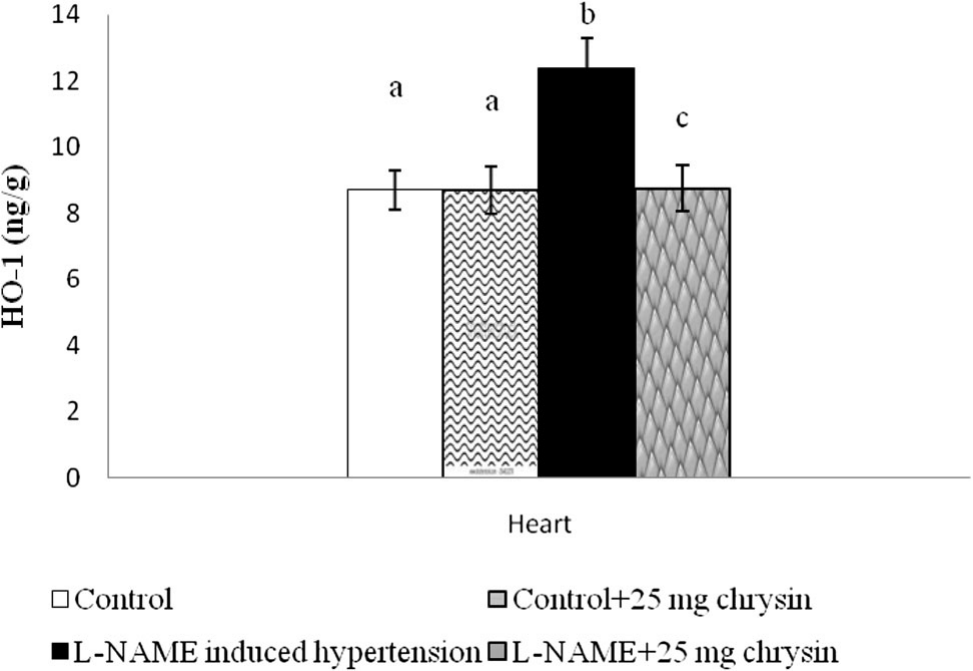
Effect of chrysin on cardiac heme oxygenase activities of experimental rats. Columns are mean ± S.D. for six rats in each group. Columns not sharing common superscript are significant with each other at *P* < 0.05 (Duncan’s multiple range test)

Figure 5 shows elevated HO-1 activity in cardiac tissue by L-NAME. However, the activity of HO-1 was significantly altered in the chrysin-treated group than in the L-NAME (*P* < 0.05) groups. There are no changes between group I and II. Chrysin (25 mg/kg of B.W) is effective dose for all parameters significant effect in L-NAME induced rats as compared to control rats. Chrysin in normal rats didn’t show any significant.

## Discussion

Chronic inhibition of NO produces volume-dependent elevation of BP; and its physiological and pathological characteristics resemble essential hypertension and very well known about acute inhibition of NO biosynthesis by in vivo supplementation of L-NAME, an l-arginine analog, leads to arterial hypertension and vasoconstriction [21]. With causes of hypertension cardiovascular damage and function impairment, in humans can be mimicked from L-NAME hypertensive rat [22]. In our results L-NAME rat heart showed a decreased maximum + LVdP/dt and minimum − LVdP/dt with increased LVDP and LVEDP when compared to control rats. The major pathogenic feature of cardiac remodelling is cardiac contractile function impairment. In prophylactical and therapeutically way chrysin prevented these structural and functional changes in the isolated heart of our study model rats. In this study, the structural and functional changes in NO-deficient rats heart hemodynamically in terms of improvement in left ventricular functions as evidenced by amelioration of Maximum + dP/dt and Maximum − dP/dt impairment that caused by L-NAME induced left ventricle hypertrophy. Further, it also helped L-NAME inducing increased LVDP and LVEDP which also reflects restoration in ventricular function. The isolated heart Langendorff study depicts that induces ventricular dysfunction induced by hypertension. It was previously known that, excess production and accumulation of extracellular matrix structural proteins, or fibrosis, resulted in- enhanced stiffness of the myocardium and impedance of ventricular contraction and relaxation, causing distorted architecture and function of the heart [23].

Oral administration of chrysin prevented and reversed LVDP, LVEDP, Maximum + dP/dt and Maximum − dP/dt changes in L-NAME hypertensive rats. The pharmacological mechanism is that chrysin inhibits generation of superoxide and hydroxyl free radicals in both enzymatic and nonenzymatic systems and evidenced by upregulation of RAS in current study. A significant elevation of cardiac NADPH oxidase activity was observed in the L-NAME group. The BP ascending effect of L-NAME in this study is associated with a significant decrease of NO along with an increase of stress oxidative biomarkers, including NADPH oxidase. These findings suggest that the BP raising effects of L-NAME only attributed to the inhibition of NO synthase but may involve oxidative stress via the activation of NADPH oxidase expression. Showing consistent with previous researcher studies, the another study, reported that L-NAME administration at a dose of 0.7 mg/ml in drinking water for 2 weeks enhanced NADPH oxidase expression in aortic tissue. Toba et al, [24] reported that L-NAME administered rats with increased oxidative stress, vascular inflammation and ACE activity and expression. In hypertensive rats L-NAME may increase blood pressure via the activation of the RAS. This finding was coincident with Zanchi et al, [25], who reported the same impact that L-NAME might increase blood pressure via activation of the RAS. We also accepted the previous researchers’ studies because the same mechanism is also found to our current study. The chrysin dose (25 mg/kg) modulated the systolic and diastolic blood pressure to elevate in rats that were co-treated with L-NAME. The significant reduction NADPH oxidase activities cardiac tissue and an increase of plasma NO with respect of chrysin, may results of reduction of systolic and diastolic BP.

In our studies shows increase in plasma Ang-II was found in L-NAME hypertensive rats compared to the level in control rats. Ang-II, the major bioactive peptide of the RAS, plays a major role in the regulation of vascular function and structure. Activation of the RAS is leading to cause hypertension in the L-NAME-induced hypertension model, which is same as previous reports [26]. Furthermore, there is an occurrence of evaluated levels of plasma renin activity, plasma Ang-II, and renal renin mRNA following L-NAME treatment [27]. The present study demonstrated that chronic L-NAME administration is associated with increased renal vasoconstriction with respect to Ang-II. As previously said RAS plays a crucial role in development of hypertension, being a potent vasoconstrictor Ang-II can activates sympathetic nerve function; also main causes of heart and vascular remodeling and heart failure in models of hypertension [28]. It is evident that NO-deficient hypertension causes an imbalance of RAS in L-NAME hypertensive rats [29]. In those rats, enhance the contractile the action of Ang-II type 1 receptor (AT1R). The authors proposed that this Ang -II hypersensitivity in L-NAME-hypertensive rats may be related to increased AT1R expression [30]. Administration of chrysin reduced plasma Ang-II level compared to untreated rats. Chrysin is already proved free radical scavenging activity and increased plasma NO activity. It may leads to chrysin reduced the plasma Ang -II and regulate the RAS system. But Chrysin did not affect the control rats.

In our studies shows reduced cGMP level in cardiac and aortic tissues of L-NAME induced hypertensive rats. Ang-II-AT1R-mediated pathway and other overlapping signaling pathways are responsible for the developing of cardiomyocyte hypertrophy. In addition of mediating immediate physiological vascular tone and BP regulation, NO attenuates hypertensive cardiac remodeling through cAMP/cGMP (cyclic adenosine monophosphate/cyclic guanosine monophosphate) mediated pathway [31]. cAMP is generated by adenylyl cyclase through G-protein coupled β adrenergic receptor-mediated stimulation. It activates protein kinase A (PKA), which may regulate the L-type Caþ2 channel and ryanodine receptor. In present study, it is shown that systolic and diastolic BP in L-NAME-induced hypertensive rats was significantly regulated by administration of chrysin. The altered BP with ascending plasma NO levels affects the level of cGMP through stimulation of guanylate cyclise causing relaxation of vascular smooth muscles [32]. Inspite of mediating through cGMP- dependent proteinkinase (PKG), cGMP/PKG could regulate Ca2^+^ entry inside the vascular smooth muscle cells, leading to the vascular relaxation [33]. In our experiment, we measured cGMP levels in rat aorta and heart tissues and chrysin level is founded to reverse the reduction of cGMP levels in comparison to L-NAME treated controls. It may cause by chrysin stimulate NO concentration and decrease systemic-hemodynamic effect leads to vascular relaxation.

The present study shows elevated HO-1 activity in cardiac tissue by L-NAME. HO is the initial and rate-limiting and microsomal enzyme in the pathway which degrades heme [34]. There are three isoforms of HO found in the body: HO-1, HO-2 and HO-3 [35]. However, the activity of HO-1 was significantly altered in the chrysin-treated group than in the L-NAME (*P* < 0.05) groups. The activation of HO-1 plays superficial role in reducing blood pressure by reducing Ang II-induced inflammation and NADPH oxidase-mediated oxidative stress through the production of anion superoxide [20]. The responsibility of HO-1 is leading its antihypersensitivity its production of CO, with a vasodilator effect [36]. In our study, it is observed cardiac HO-1 activity was significantly increased in rats that were treated with L-NAME and chrysin. That one is also clear statement that Chrysin having antihypertensive activity.

## Conclusion

These major findings of the present study demonstrated that chrysin possesses strong antihypertensive and antioxidant properties in L-NAME induced hypertensive rats resulting in the alteration of left ventricular functions, NADPH oxidase, Ho-1, Plasma ang II and higher cGMP concentration aortic and cardiac tissues. In future chrysin could effectively prevent the L-NAME hypertension and offers significant vascular and cardiac protection.

## Abbreviation

cGMP: Cyclic guanosine monophosphate
L-NAME N^ω^: Nitro-l-arginine methyl ester
LVDP: Left ventricular developed pressure
LVEDP: Left ventricular end developed pressure
